# Triple selectin knockout (ELP-/-) mice fail to develop OVA-induced acute asthma phenotype

**DOI:** 10.1186/1476-9255-8-19

**Published:** 2011-08-11

**Authors:** Ena Ray Banerjee

**Affiliations:** 1 Division of Hematology, Department of Medicine, University of Washington, 1959 NE Pacific Street, Seattle, WA 98195, USA; 2 Immunology and Regenerative Medicine Research Laboratory, Dept of Zoology, University of Calcutta, 35 Ballygunge Circular Road, Kolkata-700019, India

**Keywords:** Selectin, asthma, inflammation

## Abstract

**Objective:**

The recruitment of leukocytes from circulation to sites of inflammation requires several families of adhesion molecules among which are selectins expressed on a variety of cells. In addition, they have also been shown to play key roles in the activation of cells in inflammation.

**Methods:**

To explore the collective role of E-, L-, and P- selectins in OVA-induced Th2 mediated response in acute asthma pathophysiology, ELP-/- mice were used and compared with age-matched wildtype (WT).

**Results:**

Asthma phenotype was assessed by measuring pulmonary function, inflammation and OVA-specific serum IgE, which were completely abrogated in ELP-/- mice. Adoptive transfer of sensitized L selectin+CD4+ T cells into naïve ELP-/- mice which post-OVA challenge, developed asthma, suggesting that L-selectin may be critically involved in the onset of Th2 response in asthma. Tissue resident ELP-deficient cells were otherwise functionally competent as proved by normal proliferative response. *Conclusions*: Comparative studies between ELP-/- and WT mice uncovered functional roles of these three integrins in inflammatory response in allergic asthma. All three selectins seem to impede inflammatory migration while only L-selectin also possibly regulates activation of specific T cell subsets in lung and airways.

## Introduction

The respiratory drug market is dominated by asthma and its exacerbations (worsening symptoms, rescue medication use, and emergency department visits or hospitalizations). Asthma is the third leading cause of death in both developed and developing countries and annual direct and indirect cost of healthcare is more than $50 billion in the US alone.. mortality A small percent of non-responders (10%) account for greater than 50% of health care costs and it is for these and other patients with exacerbations that alternative target redressal is not just necessary but indispensible given the health care costs [1].

In particular, there is a need to develop drugs that control the underlying inflammatory and destructive processes. Rational treatment depends on understanding the underlying disease process and there have been recent advances in understanding the cellular and molecular mechanisms that may be involved to look for better drug targets[2]. Inflammation is key to etiology of most respiratory disorders and there is a fine balance between the beneficial effects of inflammation cascades and inflammation cascades lead to development of diseases such as chronic asthma, rheumatoid arthritis, psoriasis, multiple sclerosis and inflammatory bowel disease. The specific characteristics of inflammatory response in each disease and site of inflammation may differ but recruitment and activation of inflammatory cells and changes in structural cells remain a universal feature along with a concomitant increase in the expression of components of inflammatory cascade including cytokines, chemokines, growth factors, enzymes, receptors, adhesion molecules and other biochemical mediators [3].

The chronic airway inflammation of asthma is unique in that the airway wall is infiltrated by T lymphocytes of the T-helper (Th) type 2 phenotype, eosinophils, macrophages/monocytes and mast cells. Accumulation of inflammatory cells in the lung and airways, epithelial desquamation, goblet cell hyperplasia, mucus hypersecretion and thickening of submucosa resulting in bronchoconstriction and airway hyperresponsiveness are important features of asthma [4] Both cells from among the circulating leukocytes such as Th2 lymphocytes, mature plasma cells expressing IgE, eosinophils [5]and neutrophils as well as local resident and structural cells constituting the 'respiratory membrane' (airway epithelial cells, fibroblasts, resident macrophages, bronchial smooth muscle cells, mast cells etc.) contribute to the pathogenesis of asthma [6]. Cross-linking of IgE receptors on mast cells releases histamines, prostaglandins, thromboxane and leukotrienes leading to bronchoconstriction, vasodilation and mucus secretion. A cascade of interactions between cells and soluble molecules result in bronchial mucosal inflammation and lead to airway hyperresponsiveness [7].

Leukocyte emigration into lung is an important event in the pathogenesis of asthma, likely mediated by a series of leukocyte adhesion molecule interactions with endothelium of which the various ICAMs, adhesion molecules [8] and selectins [9,10] have been found to be critically important. Among the integrins, α4 is key in initial signaling for sensitization as well as migration for the onset and development of a full blown acute asthma phenotype as well as airway remodeling in chronic asthma while β2 integrins are solely required for mechanical migration of leukocytes [11,12].

During inflammatory recruitment in lung and airways, the initial contact of leukocytes with the endothelium is mediated by selectins and their ligands inducing the rolling of leukocytes along the vessel wall [13-17] . This rolling phenomenon is a pre-requisite for the subsequent firm adhesion and transmigration, which is mediated by members of the integrin family, e.g. β2 integrins, and immunoglobulin gene superfamily, e.g. intercellular adhesion molecule-1 (ICAM-1) [18-20].Peribronchial inflammation contributes to the pathophysiology of allergic asthma. In many vascular beds, adhesive interactions between leukocytes and the endothelial surface initiate the recruitment of circulating cells. Such movement is believed to follow a coordinated and sequential molecular cascade initiated, in part, by the three members of the selectin family of carbohydrate-binding proteins: E-selectin (CD62E), L-selectin (CD62L) and P-selectin (CD62P).

The role of selectins in neutrophil trafficking in the lungs was frequently considered negligible since the narrow pulmonary capillaries cannot accommodate the typical selectin-mediated rolling phenomenon. Selectins especially did not seem necessary in lung neutrophil sequestration since the deceleration of circulating neutrophils prior to their firm adherence was effectively achieved by their mechanical retention[10]. Yet a large body of experimental data demonstrating that selectin inhibition (via the use of blocking antibodies or selectin antagonists or transgenic knockout of 1 or more selectins) frequently protected animals from Acute lung injury. [Subset-Specific Reductions in Lung Lymphocyte Accumulation Following intratracheal antigen Challenge in Endothelial Selectin-Deficient Mice [21-23]. P-selectin mediated platelet-neutrophil interactions are critical to the development of ALI, to which they contribute by enhancing their respective activations, which trigger the production of TXA2 and other inflammatory mediators [24].

In addition to being responsible for the rolling phenomenon, selectins have been implicated as important signaling molecules involved in leukocyte activation as well. The selectin gene family closely linked on mouse chr1, encodes 3 structurally related proteins that display differential spatial and temporal expression within the vascular system. Endotherlial cells express E-selectin, platlets express P-selectins and leukocytes express L-selectin. Whereas P-selectin (CD62P) is rapidly mobilized to the surface of activated endothelium or platlets, E-selectin (CD62E) expression is induced by inflammatory cytokines. L-selectin (CD62L) is constitutively expressed on most leukocytes.

Selectin expression can be up-regulated under inflammatory conditions in experimental animals [25-28] and humans [29]. Although there appear to be some variations in E-selectin expression depending on the disease state, the general consensus from these studies is that E-selectin is most strongly expressed on endothelial cells of portal tract vessels and hepatic venules and to a lesser degree on sinusoidal lining cells[25]. In contrast, L-selectin is constitutively present on most types of leukocytes [30]. *In vitro *studies showed that E-selectin on endothelial cells can induce up-regulation of Mac-1 (CD11b/CD18) on neutrophils[31]. In addition, L-selectin ligation or crosslinking induced up-regulation of Mac-1 and priming for superoxide formation [19]. Thus, E-, P-, and L-selectin may have an important function in Th2 response in OVA-induced asthma.

The objective of this study was to investigate the functional significance of E-, P- and L-selectin in an experimental model of OVA-induced lung injury. OVA induce a Th2-mediated inflammatory response characterized by inflammatory recruitment in lungs and airways, mucus over-secretion and airways hyperresponsiveness. Our previous studies [11,12] have shown the involvement of various families of adhesion molecules viz. α4β1, β2 and VCAM-1 facilitate leukocyte transmigration, adherence to parenchymal cells, and Th2 response, in the pathophysiology of various inflammatory disease models such as allergic asthma and aseptic peritonitis. This study elucidates the effect of deletion of all three selectins in the development of allergic asthma phenotype in mice.

## Materials and methods

### Animals

C57BL6 mice were used as described previously [11,12]. Wildtype (WT) and ELP-/- (was kindly donated by Richard O. Hynes of the Howard Hughes Medical Institute and Center for Cancer research, MIT, Cambridge, MA) both on a C57BL6 background were used. All animals were maintained under SPF conditions in the animal facility of the University of Washington following strict guidelines laid down by IACUC. (n = 5 mice per experimental group).

### Allergen sensitization and challenge

**Figure 1:**
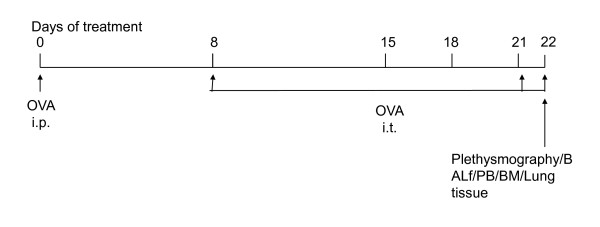
**Study protocol for development of acute allergic asthma disease model in mice**.

Mice were sensitized and later challenged with OVA (Pierce, Rockford, IL) as described previously [11,12]. Mice were immunized with OVA (100 mg) complexed with aluminium sulfate in a 0.2-ml volume, administered by i.p. injection on day 0. On days 8 (250 mg of OVA) and on days 15, 18, and 21 (125 mg of OVA), mice were anesthetized briefly with inhalation of isoflurane in a standard anesthesia chamber and given OVA by intratracheal (i.t.) administration. Intratracheal challenges were done as described previously (Iwata A, J Immunol. 2003;170:3386). Mice were anesthetised and placed in a supine position on the board. The animal's tongue was extended with lined forceps and 50 ml of OVA(in the required concentration) was placed at the back of its tongue. The control goup received normal saline with aluminium sulfate by i.p. route on day 0 and 0.05 ml of 0.9% saline by i.t. route on days 8, 15, 18, and 21 (Figure 1).

### Pulmonary function test

*In vivo *airway hyperresponsiveness to methacholine was measured 24 hours after the last OVA challenge by both invasive and non-invasive plethysmography.

### Invasive plethysmography

On d 22, 24 h after the last intra-tracheal allergen (OVA) challenge invasive pulmonary mechanics were measured in mice in response to methacholine in the same manner as previously described [11] with the following modifications: a) the thorax was not opened, b) mice were ventilated with a tidal volume of 200 μl and respiratory rate of 120 breaths/min using a MiniVent Ventilator for Mice (Harvard Apparatus, Holliston, MA), c) mice received aerosolized solutions of methacholine (0, 3.125, 6.25, 12.5, 25, 50, and 100 mg/ml in normal saline) via an AER 1021 nebulizer aerosol system (Buxco Electronics, Inc., Wilmington, NC) with 2.5-4 micron aerosol particle size generated by NEB0126 nebulizer head (Nektar Therapeutics, San Carlos, CA), and d) a commercial plethysmography system (Model PLY4111 plethysmograph, MAX II amplifier and pressure transducer system, and Biosystem XA software, Buxco Electronics, Inc.) was used to determine R_L _as calculated from measures of pressure and flow and expressed as cmH_2_O/ml/s). Non-invasive plethysmography (expressed as Penh) was also assessed on d 22 in independent experiments.

**Non-invasive whole body plethysmography **in conscious, free moving, spontaneously breathing mice using whole-body plethysmography (model PLY 3211; Buxco Electronics, Sharon, CT) as previously described [41]. Mice were challenged with aerosolized saline or increasing doses of methacholine (5, 20, and 40 mg/ml) generated by an ultrasonic nebulizer (DeVilbiss Health Care, Somerset, PA) for 2 min. The degree of bronchoconstriction was expressed as enhanced pause(P_enh_), a calculated dimensionless value, which correlates with the measurement of airway resistance, impedence, and intrapleural pressure in the same mouse. P_enh _readings were taken and avergared for 4 min after each nebulization challenge. Penh was calculated as follows: P_enh_=[(Te/Tr-1)X (PEF/PIF), where T_e _is expiration time, T_r _is relaxation time, PEF is peak expiratory flow, and PIF is peak inspiratoy flow × 0.67 co-efficient. The time for the box pressure to change from a maximum to a user-defined percentage of the maximum represents the relaxation time. The T_r _measurement begins at the maximum box pressure and ends at 40%.

### Cell suspensions prepared from tissues

After pulmonary function testing, the mouse underwent exsanguination by intra-orbital arterial bleeding and then BALF (0.4 ml three times) of both lungs. Total BALF fluid cells were counted from a 50 ml aliquot and the remaining fluid was centifuged at 200 *g *for 10 min at 4°C and the supernatants stored at -70°C for assay of BALF cytokines later. The cell pellets were resuspended in FCS and smears were made on glass slides. The cells, after air drying, were stained with Wright-Giemsa (Biochemical Sciences Inc, Swedesboro, NJ) and their differential count was taken under a light microscope at 40X magnification. Cell number refers to that obtained from lavage of both lungs/mouse. Lung parenchyma was prepared in the following way: lung mincing and digestion was performed after lavage as described previously [13] with 100 u/ml collagenase for 1 hr at 37°C, and filtered through a 60# sieve (Sigma). All numbers mentioned in this paper refer to cell sobtained from one lung/mouse.

### Lung histology

Lungs of other animals of same group were were fixed in 4% paraformaldehyde overnight at 4°C. The tissues were embedded in paraffin and cut into 5 mm sections. A minimum of 15 fields were examined by light microscopy. The intensity of cellular infiltration around pulmonary blood vessels was assessed by Hematoxylin and Eosin staining. Airway mucus was identified by staining with Alcian blue and Periodic Acid Schiff staining as described previously [11,12]

### Smear evaluation

Proportions of eosinophils and mast cells were assessed in cytospin smears stained with Hematoxylin and Eosin by Diff Quik stain from Fisher.

### Fluorescin-activated cell sorter (FACS) analysis

Cells from hemolysed peripheral blood (PB), bone marrow(BM), bronchoalveolar lavage (BALF), lung parenchyma (LP), and spleen were analyzed on a FACSCalibur (BD Immunocytometry Systems, San Jose, CA) by using the CELLQuest program. Staining was performed by using antibodies conjugated to fluorescin isothiocyanate (FITC), phycoerythrin (PE), allophucocyanin (APC), Peridinin Chlorophyll Protein (Per CP-Cy5.5) and Cy-chrome (PE-Cy5 and PE-Cy7). The following BD pharmingen (San Diego, CA) antibodies were used for cell surface staining: APC-conjugated CD45 (30F-11), FITC-conjugated CD3(145-2C11), PE-Cy5 conjugated CD4 (RM4-5), PE-conjugated CD45RC (DNL-1.9), APC-conjugated CD8(53-6.7), PE-Cy5 conjugated B220 (RA3-6B2), FITC-conjugated IgM, PE-conjugated CD19 (ID3), PE-conjugated CD21(7G6), FITC-conjugated CD23 (B3B4), APC-conjugated GR-1(RB6-8C5), and PE-conjugated Mac1(M1/70). PE-Cy5 conjugated F4/80 (Cl:A3-1(F4/80)) was obtained from Serotec Ltd., Oxford, UK.

### CFU-c assay

To quantitate committed progenitors of all lineages, CFU-C assays were performed using methylcellulose semisolid media (Stemgenix, Amherst, N.Y.) supplemented with an additional 50 ng of stem cell factor (Peprotech, Rocky Hill, N.J.) per ml. Next, 50,000 cells from bone marrow, 500,000 cells from spleen, 0.01 million cells from lung and BALF, and 10 ml peripheral blood were plated on duplicate 35-mm culture dishes and then incubated at 37°C in a 5% CO_2_-95% air mixture in a humidified chamber for 7 days. Colonies generated by that time were counted by using a dissecting microscope, and all colony types (i.e., burst forming units-erythroid [BFU-e], CFU-granulocyte-macrophage [CFU-GM], and CFU-mixed [CFU-GEMM]) were pooled and reported as total CFU-C. Total CFU-c per organ was calculated by extrapolating CFU-c against number of plated cells to the total number of cells in the organ.

### ELISA for cytokines

Th2 cytokines (IL-4 and 5) and TNFa and IFNg in BALF and serum (previously frozen at -70°C) were assayed with mouse Th1/Th2 cytokine CBA (BD Biosciences, San Diego, CA) following the manufacturer's protocol. According to the manufacturer's protocol. IL-13 and Eotaxin were measured by Quantikine M kits from R&D Systems, Minneapolis, MN.

### ELISPOT

IL-4+ and IFN-γ+ cells in single cell suspensions from lung parenchyma and BALf were detected employing standard ELIspot assays (Lee S-H, Nat Med. 2003;9:1281) using detection and capture monoclonal antibodies and AEC substrate reagent from BD Biosciences. Dots were counted manually using 40X magnification.

### OVA specific IgE, and IgG1 in serum

Anti-mouse IgE (R35-72) and IgG1(A85-1) from BD Biosciences, San Diego, CA were used for measuring OVA specific IgE and IgG1 (in serum previously frozen at -70°C) respectively by standard ELISA procedures as previously described [15].

### T cell proliferation assay

MACS-separated CD4þ and CD8þ T cells from spleens were stimulated in vitro with various concentrations of stimuli (CD3/CD28, phorbol myristic acetate (PMA)/ionomycin, irradiated antigen-presenting cells (APCs), and lipopolysaccharide (LPS) to assay proliferative responses. After 72 hours, proliferation was measured by CellTiter96 assay from Promega (Madison, WI, USA) measuring OD at 570 nm.

### Adoptive transfer

At day 8 after i.p. sensitization, 5 × 10^6 ^CD4+ splenocytes from both WT controls or ELP-/- mice were purified by magnetic-activated cell sorting (MACS, Miltenyi Biotec, Auburn, CA, USA), then injected into the tail veins of na""ve controls or ELP-/- recipients. The mice were subsequently challenged with 3 i.t. instillations of OVA over the next 72 hours, and sacrificed 24 hours after the last instillation [14]. For negative control (Gr#4), CD62L-CD4+ T cells were separated by MACS from ELP-/- donors. Gr#1 is the positive control here.

### Statistical analysis

Statistical differences among samples were tested by Student *t *test. *P *value less than 0.05 was considered statistically significant.

## Results

### Complete abrogation of composite asthma phenotype in KO mice

**Figure 2:**
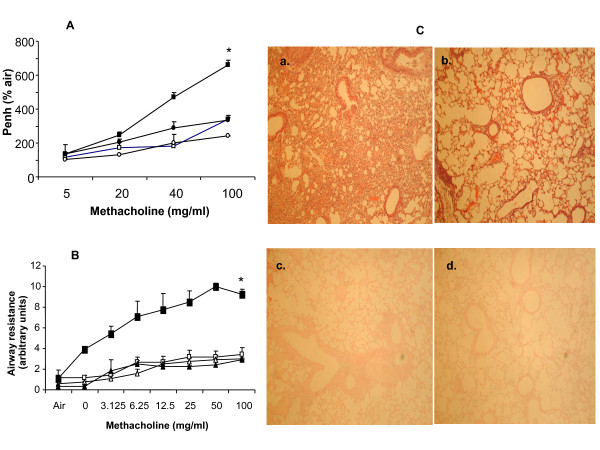
**Non-development of composite asthma phenotype in ELP-/- mice**. **Figure 2A. Pulmonary function test by non-invasive whole body plethysmography**. Functional response to increasing doses of methacholine show no increase in response from baseline in ELP-/- mice in comparison with WT post-OVA. WT+alum (white square), WT+OVA (black square), ELP-/-+alum (white square) and ELP-/-+OVA (black square). **Figure 2B. Pulmonary function test by invasive plethysmography**. Functional response to increasing doses of methacholine show no increase in response from baseline in ELP-/- mice in comparison with WT post-OVA. [WA = WT with alum and saline treatment, EA = ELP-/- mice same placebo treatment, WO = WT sensitized and challenged with OVA, EO = OVA treatment with ELP-/- mice.] WA (white square), WO (black square), EA (white triangle), EO (black triangle). **Figure 2C. Lung histology. Panel a, b**. H&E staining of paraffin lung sections. a. WT+OVA, b. ELP-/-+OVA; **Panel c, d**. Alcian blue staining of paraffin lung sections. C. WT+OVA, d. ELP-/-+OVA.

Our first aim was to find out whether composite asthma phenotype developed (Figure 1) in the absence of the triple selctins in a knockout mouse which should indicate what role they have in the onset of the disease pathophysiology. We found that following allergen challenge, compared to the WT mice which develop all the hallmarks of the composite asthma phenotype, the yardsticks quantified in the ELP-/- mice were all similar to the saline treated baseline controls (Figure 2A-C) viz. there was no significant increase in either Penh or R_L _in response to increasing doses of aerosolized methacholine as measured by a plethysmometer nor inflammation in the airways or lung parenchyma as detected H&E staining of histological sections of the lung post-OVA in the ELP-/- mice vs. WT.

### Inhibition of inflammatory recruitment in airways despite peripheral leukocytosis

Once it was clear that the preclinical parameters for acute allergic asthma failed to develop in the knockout mosue, we embarked on dissecting the patterns of inflammatory recruitment from bone marrow to peripheral blood to lung parenchyma to the lung interstitium which is the stage for the drama of the disease pathology to manifest itself. Table 1A-C show that despite increased number of mature leukocytes and progenitors in BM and circulation of post-OVA ELP-/- mice, their number was insignificant especially in the airways. Lung of post-OVA ELP-/- mice show elevated number of monocytes, macrophages and neutrophils (myeloid population) but no eosinophils and mast cells as opposed to the higher number of these cells in OVA-treated WT. (Table 1, 2 and 3).

**Table 1:** Total number of cells (×10^6^) in various tissues.

	**BM/femur**	**PB/ml**	**Spleen**	**BALf/2lungs**	**LP/2lungs**
WT+alum	14.28 ± 2.38	4.17 ± 1.01	103.14 ± 31.95	0.68 ± 0.03	1.8 ± 0.63
WT+OVA	31.35 ± 11.95	7.36 ± 2.01	167.45 ± 45.97	8.4 ± 2.22	3.14 ± 0.95
ELP-/-+alum	29.54 ± 2.08*	15.31 ± 0.41*	280.43 ± 38.57*	0.78 ± 0.005	3.79 ± 0.07*
ELP-/-+OVA	33.18 ± 1.06*	15.5 ± 0.77*	247.63 ± 10.36*	0.81 ± 0.013*	5.6 ± 0.17*

Total number of cells (x10^6^) in different tissues;

**Table 2:** Total number of different types of leukocytes (x10^6^)in various tissues.

**BM/femur**	**Mononuclear**	**PMN**	**Eos**			
WT+alum	8.61 ± 1.92	5.13 ± 1.56	0.53 ± 0.02			
WT+OVA	14.68 ± 3.79	11.48 ± 3.3	5.17 ± 1.22			
ELP-/-+alum	19.27 ± 2.48*	9.53 ± 1.02*	0.73 ± 0.06			
ELP-/-+OVA	19.56 ± 5.59¶	13.3 ± 4.11¶	0.31 ± 0.04¶			
**PB/ml**	**Lymphocyte**	**Monocyte**	**Basophil**	**PMN**	**Eosinophil**	
WT+alum	2.68 ± 0.16	0.14 ± 0.06	0.03 ± 0.009	1.17 ± 0.28	0.12 ± 0.01	
WT+OVA	2.39 ± 0.43	1.6 ± 0.13	0.11 ± 0.003	1.34 ± 0.36	1.9 ± 0.25	
ELP-/-+alum	9.67 ± 2.01*	0.57 ± 0.03*	0.07 ± 0.004	2.41 ± 0.56*	0.17 ± 1.01	
ELP-/-+OVA	9.21 ± 1.09¶	0.49 ± 0.12¶	0.08 ± 0.002¶	5.45 ± 0.38¶	0.25 ± 0.07¶	
**BALf/2lungs**	**Lymphocyte**	**Monocyte**	**Macrophage**	**PMN**	**Eosinophil**	**Mast cell**
WT+alum	Entirely epithelial cells and monocytes/macrophages					
WT+OVA	1.93 ± 0.38	1.17 ± 0.19	2.11 ± 0.12	1.61 ± 0.33	0.03 ± 0.003	0.033 ± 0.001
ELP-/-+alum	Only epithelial cells and monocytes/macrophages					
ELP-/-+OVA	Only epithelial cells and monocytes/macrophages					
**LP/2lungs**	**Lymphocyte**	**Monocyte**	**Macrophage**	**PMN**	**Eosinophil**	**Mast cell**
WT+alum	0.2 ± 0.09	0.8 ± 0.02	0.48 ± 0.32	0.3 ± 0.09	0	0
WT+OVA	0.72 ± 0.04	0.44 ± 0.11	0.79 ± 0.11	0.6 ± 0.03	0.57 ± 0.13	0.012 ± 0.001
ELP-/-+alum	0.41 ± 0.02*	1.65 ± 0.35*	1.07 ± 0.04*	0.65 ± 0.02	0	0
ELP-/-+OVA	0.51 ± 0.03	2.01 ± 0.41¶	1.22 ± 0.37¶	1.35 ± 0.03¶	0	0

Number of cells (× 10^6^) in various cell subsets in different tissues

**Table 3:** Total number of CFU-c in various tissues.

	**BM/femur**	**PB/ml**	**BALf/2 lungs**	**LP/2 lungs**
WT+alum	45892 ± 1541	160 ± 34	3 ± 0.2	59 ± 12
WT+OVA	76894 ± 1376	1020 ± 69	872 ± 37	850 ± 14
ELP-/-+alum	61268 ± 3497*	740 ± 45*	11 ± 5*	121 ± 47*
ELP-/-+OVA	63550 ± 3492	845 ± 117¶	14 ± 6.3¶	269 ± 68¶

Number of progenitor cells in various tissues evaluated by CFU-c. The numbers denoting progenitors have been rounded to the nearest whole number. n = 5/group. * denotes p value < 0.05 compared to WT+alum and ¶ denotes p value < 0.05 compared to WT+OVA.

### ELP-/- B cells are incapable of sequestering either total or OVA-specific Igs

**Figure 3:**
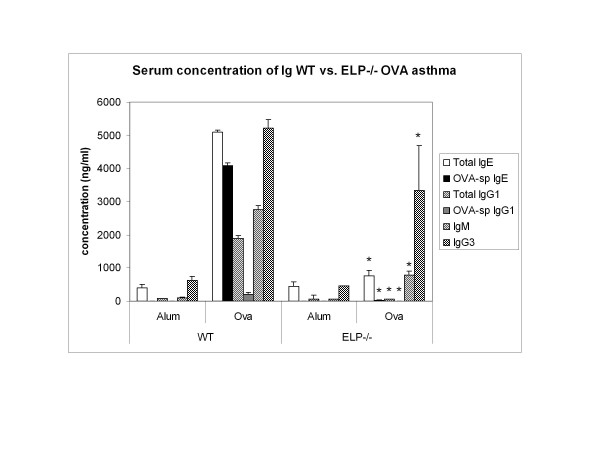
**Serum concentration of immunoglobulins in ELP-/- vs**. WT post-OVA. Serum from infra-orbital bleeding of the experimental mice, collected in heparinized tubes, were frozen at -70°C and later methods described in [41] were used to detect total and OVA-specific immunoglobulin concentration. Presented values are data pooled from 2 independent experiments with 5 mice per experimental group measured in dulicate in 96-well format. * denotes p value < 0.05 compared to WT+OVA values.

Since overall allergy depends on cross talk between the T and B cells and a concerted and sequential interplay between the two cell populations, our next question was whether the knockout B cells themselves were otherwise functionally competent? B cell function was tested by assaying total as well as OVA-specific Igs post OVA. Figure 3 shows that other than total IgG3, all other Igs, notably IgE and IgG1 were significantly inhibited and even IgG3 was decreased compared to WT+OVA values.

### Lack of inflammatory and Th2 response in KO mice

**Figure 4:**
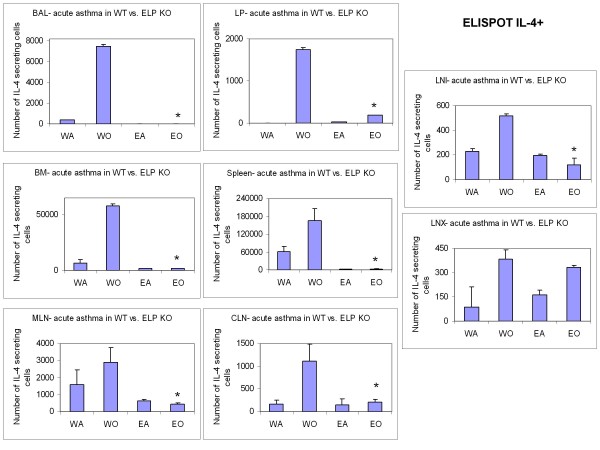
**ELISPOT of Th2 cells in lymphoid tissues**. cells.. Number of IL-4+ cells averaged from triplicate wells of a 96-well ELISPOT plate ± SEM of 2 independent experiments. * denotes p value < 0.01 compared to WT+OVA.

**Figure 5:**
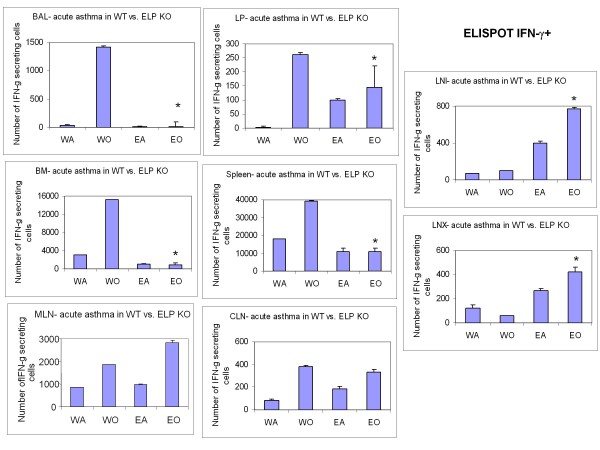
**ELISPOT of Th1 cells in lymphoid tissues**. cells.. Number of IFN-γ+ cells averaged from triplicate wells of a 96-well ELISPOT plate ± SEM of 2 independent experiments. * denotes p value < 0.01 compared to WT+OVA.

Following the analyses of the cells taking part in the development of the allergic inflammation itself, both locally and systemically, the next question to be addressed was the actual amount of the cytokine mediators that were being synthesized and released by the inflammatory cells both for propelling their recruitment in the relevant sites as well as their specific functional manifestation therein. Release of Th2 specific cytokines was significantly downregulated in KO BALf and plasma (Table 3) compared to OVA-treated control. Detection of virtually no IL-4-secreting cells in all except LNX (BALf, LP, spleen, BM, MLN, CLN, LNI) and very low number of IFN-γ-secreting cells in all but the peripheral lymph nodes (CLN, LNX, and LNI) was found (Table 4 Figure 4, 5).

**Table 4:** Cytokine concentration (pg/ml) in plasma and BALf post-OVA in WT vs.

**Plasma**	**Mean**	
	**WT**	**ELP-/-**
**IL-2**	41.6 ± 7	4.95 ± 1.75*
**IFN-γ**	40.89 ± 1.19	2.86 ± 0.88*
**TNF-α**	9.1 ± 0.4	nd
**IL-10**	78.05 ± 22.65	6.55 ± 3.05*
**IL-4**	81.95 ± 0.85	nd
**IL-5**	244.8 ± 22.8	87.7 ± 6.6*
**IL-13**	136.1 ± 0.8	nd
**Eotaxin**	337.33 ± 40.32	nd
**MMP-9**	96696.45 ± 158.11	70603 ± 40399.7*
**SDF-1α**	665.6 ± 73.7	48.65 ± 7.55*
**KC**	23.8 ± 2	nd
**TGF-β**	19063.55 ± 4377.45	nd
		
**BALf**	**Mean**	
	**WT**	**ELP-/-**
**IL-2**	36.6 ± 0.7	38.4 ± 0.2
**IFN-γ**	144 ± 27.5	nd
**TNF-α**	20.4 ± 0.8	5.2 ± 0.6*
**IL-10**	8.7 ± 0.6	2.15 ± 0.15*
**IL-4**	94.75 ± 9.35	nd
**IL-5**	94.85 ± 13.35	10.1 ± 0.8*
**IL-13**	131.8 ± 3.9	9.1 ± 8.3*
**Eotaxin**	431 ± 84.7	12 ± 0.2*
**MMP-9**	945.3 ± 23.9	371.05 ± 67.25*
**SDF-1α**	260.8 ± 17.5	35.75 ± 2.95*
**KC**	17.9 ± 0.6	6.95 ± 0.45*
**TGF-β**	467 ± 15.7	nd

ELP-/-.

nd = not detected

The concentration of cytokines was measured by outsourcing to Pierce, Endogen by multiplexing with their patented technology. Presented data evaluation was outsourced to Pierce Endogen. Searchlight™ technology was used to assay the following cytokines in triplicate and data presented is mean of samples from 5 mice per group and pooled from 2 independent experiments ± SEM. Other than IL-2 all Th1 and Th2 as well as other associated cytokines levels were decreased in KO BALf while a universal downregulation was found in all cytokines in plasma. Presented data is average of triplicate of samples (n = 5/group) ± SEM of 2 independent experiments. *p value < 0.01 compared to WT+alum (baseline) values.

### ELP-/- T cells retain normal proliferative response to mitogenic stimuli

**Figure 6:**
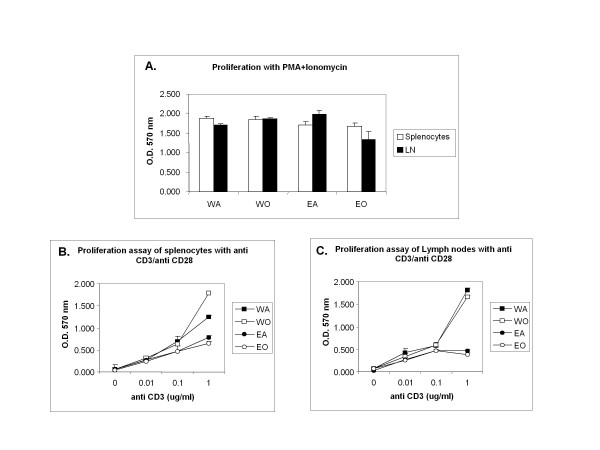
**Functional tests for T cells**. Unseparated T cells from spleen and peripheral lymph nodes (pooled cervical and axillary lymph nodes) were evaluated for proliferative responses to various stimuli and readout taken at 570 nm following MTT assay (Promega) **A**. Proliferation assay with PMA+ionomycin; **B**. Proliefration to anti CD3/CD28 with cells from spleen, and **C**. with cells from the same lymph nodes as above.

If the cytokine release by the knockout Th2 cells were unsatisfactory as we found, the next querry to be addressed was, were they functionally competent otherwise? To assess the functional status of KO T cells, CD4+ T cells were isolated from spleen and peripheral lymph nodes and subjected to proliferation assays to various stimuli (PMA, ionomycin and anti CD3/CD28) which showed efficient proliferative response similar to WT (Figure 6A-C). In addition, CD3 level on the T cells in WT and ELP-/- cells before and after OVA are similar (Table 4) and no difference in T cytokine production by ELISPOT was observed (data not presented).

### Adoptive transfer of CD62L+CD4+ splenocytes from sensitized WT to naïve ELP-/- recipients could reverse inhibition of asthma development

**Figure 7:**
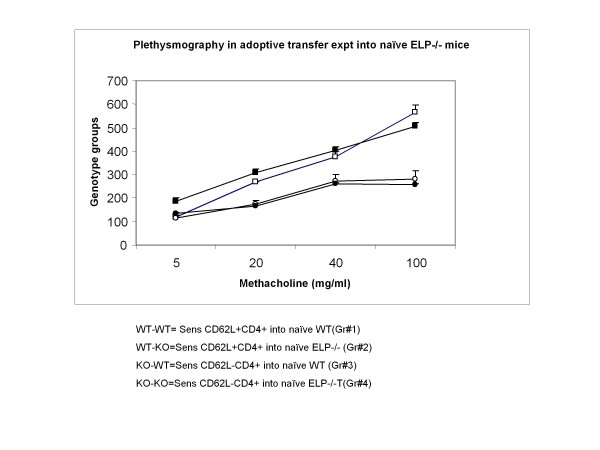
**Non-invasive whole body plethysmography to assess pulmonary function in adoptively transferred recipients**. **(**n = 4 per group). [Refer Table 2 for adoptive transfer groups.] Gr#1 (white square), Gr#2 (Black square), Gr#3 (white circle), Gr#4 (black circle).

Our next question was if as we found in all the previous quantifications, in the deleted scenario allergic asthma failed to develop, to reverse the effects of deletion, which cells will be most effective so that we may clearly delineate the role playing of that particular cell subset that is responsible for the pathology by virtue of their expression of the triple selctins, we did the following elimination experiment by various permutations and combinations. Our most obvious candidate was the CD4+ T cells. To assess whether adoptively transferred CD4+ T cells from sensitized wildtype mice, where all three selectins are in place, could reverse the non-development of asthma phenotype in ELP-/- mice, adoptive transfer of the aforementioned cells from spleen of senstitized WT mice were transferred by tail vein injection to naïve ELP-/- mice followed by OVA challenge as described in Materials and Methods. Surprisingly, we found that these ELP-/- did respond to the OVA challenge and developed a significant asthma phenotype (Gr#2) compared to either the Grs# 3 or 4 where CD4+ T cells from spleen of sensitized ELP-/- mice were used. The experiment was validated by Gr#1 (positive control). (Table 5 and Figure 7)

**Table 5:** Adoptive transfer groups.

	**Donors**	**Recipients**	**Asthma phenotype**
Gr#1	CD4+ from Sensitized WT	Naïve WT	+
Gr#2	CD4+ from Sensitized WT	Naïve ELP-/-	+
Gr#3	CD4+ from Sensitized ELP-/-	Naïve WT	-
Gr#4	CD4+ from Sensitized ELP-/-	Naïve ELP-/-	-

Donors were always sensitized mice and CD4+ T splenocytes were isolated by MACS and adoptively transferred into naïve recipients (5 × 10^6^) by tail vein injection. Splenocytes were collected a week after sensitization of donor by i.p. injection of OVA-alum. The recipients were then challenged by intra-tracheal OVA instillation over the next 72 h and sacrificed and evaluated 24 h after the last challenge.

### Functionally incompetent dendritic cells in knockout mice ruled out

We also wanted to check whether the regulatory T cells and dendritic cells were similar in the knockout mice. Table 6 shows that both WT and KO tissues showed similar expression of Treg and dendritic cells.

**Table 6:** Number of total and LECAM+ regulatory T cells and dendritic cells in various hematopoietic and non-hematopoietic tissues in WT vs.

	**Total**					
	CD45+	CD3+	CD4+	CD8+	CD4+	CD11c+
					CD25+	
**BM**						
WO	9.67 ± 2.31	0.15 ± 0.06	0.02 ± 0.001	0.12 ± 0.09	0.19 ± 0.06	0.020 ± 0.004
EO	10.16 ± 3.34	0.18 ± 0.07	0.03 ± 0.015	0.14 ± 0.03	0.18 ± 0.07	0.018 ± 0.006
**PB**						
WO	26.90 ± 4.41	9.49 ± 4.76	3.59 ± 1.05	5.90 ± 0.87	1.38 ± 0.56	1.18 ± 0.76
EO	29.99 ± 5.67	6.49 ± 2.13	2.22 ± 1.02	4.27 ± 0.56	1.05 ± 0.74	1.61 ± 0.13
**Spleen**						
WO	234.89 ± 54.87	118.69 ± 32.87	48.79 ± 11.09	69.90 ± 11.21	27.01 ±	53.91 ± 4.98
EO	212.73 ± 43.98	109.09 ± 18.24	44.76 ± 4.97	63.33 ± 14.32	30.73 ±	41.61 ± 9,61
**MLN**						
WO	38.74 ± 4.73	15.22 ± 5.52	4.85 ± 1.09	10.37 ± 2.21	4.95 ± 0.54	7.00 ± 0.43
EO	36.10 ± 9/65	17.48 ± 5.98	4.95 ± 0.67	12.53 ± 3.86	5.30 ± 0.04	7.42 ± 1.09
**CLN**						
WO	16.35 ± 3.65	4.41 ± 1.06	1.452 ± 0.04	2.89 ± 0.03	12.91 ± 0.54	2.78 ± 0.54
EO	14.00 ± 4.45	6.36 ± 0.64	5.05 ± 0.43	1.31 ± 0.54	3.51 ± 1.85	2.68 ± 0.31
**LNI**						
WO	168.37 ± 7.54	8.67 ± 2.32	2.76 ± 0.54	5.91 ± 1.09	2.82 ± 0.06	3.98 ± 0.43
EO	21.75 ± 3.85	10.53 ± 0.65	2.980.54 ±	7.55 ± 2.03	3.19 ± 1.07	4.47 ± 1.14
**LNX**						
WO	11.90 ± 3.28	5.61 ± 0.43	3.93 ± 0.54	3.68 ± 0.54	2.43 ± 0.87	2.26 ± 1.09
EO	13.06 ± 6.90	5.94 ± 1.33	4.71 ± 2.44	2.82 ± 0.98	3.28 ± 0.54	2.50 ± 0.23
**LP**						
WO	0.70069 ± 0.01	0.01962 ± 0.0030.004	0.00294 ± 0.003	0.01668 ± 0.003	0.09459 ± 0.003	0.00090 ± 0.00001
EO	0.71086 ± 0.04	0.01987 ±	0.00327 ± 0.001	0.01660 ± 0.004	0.09761 ± 0.004	0.00092 ± 0.00004

KO mice.

Cell suspension was prepared either by flushing or homogenizing tissues such as bone marrow (BM) cells were prepared by flusing femurs, PB (peripheral blood cells were prepared by hemolyzing whole blood to remove RBCs, LP (lung parenchyma) cells were prepared by homogenizing exsanguinated lung tissue and BALf (bronchoalveolar lavage fluid) cells were prepared by flushing airways in the exsangunated lung, spleen cell suspension was prepared by flushing the spleen and MLN (mesenteric lymph node), LNI (Inguinal lymph node), CLN (cervical lymph node) and LNX (axillary lymph node) cells were prepared by similar method. The cells were resuspended in 1X PBS with 5% BSA and stained with fluorochrome conjugated antibodies and quantified by flow cytometry. WO denotes Wild type mice challenged with OVA and EO denotes ELP-/- challenged with OVA. Presented data is average of triplicate of samples (n = 5/group) ± SEM of 2 independent experiments. *p value < 0.01 compared to WT+alum (baseline) values.

## Discussion

A similar protective effect as was observed in α4-/- and β2-/- mice in our earlier studies in the acute allergic asthma model, was also observed here with the ELP-/- mice [32]. This raises the question of redundancy of the roles of all these cell adhesion molecules, each cardinal in their individual capacities in cellular migration in inflammation and each affecting specific cellular components in the inflammatory cascade. So the fulcrum of the following discussion in an effort to interpret the observations described in this paper shall revolve around (a) the role played by all three selectins in context with the cells involved in the onset, development and maintenance of allergy and associated inflammation in acute asthma, (b) their role in the etiology of the disease or co-operative functions at later stages of the disease manifestation, and (c) whether pathways traversed by these adhesion molecules intersect, overlap at certain points or are indeed redundant. We shall try and address each point in our discussion of the data in the following paragraphs.

First of all, as the data in our study shows, despite increased and extensive peripheral leucocytosis, especially in eosinophils, deletion of all three selectins leads to a complete inhibition of the development of the asthma phenotype in the OVA-treated ELP-/- mouse. The failure of the eosinophils to travel firstly to the lung and thence to the airways to elicit the downstream effects of composite asthma phenotype may have been abrogated due to the absence of either E- or L- selectin or all three. Let us try and eliminate the possible candidates.

Previous studies [33] have shown a key role for P-selectin in modulating leukocyte behavior, e.g. ragweed induced recruitment of eosinophils to the peritoneum suggests a crucial role for P-selectin in Eos recruitment. Neither E- nor L-selectin appear to mediate leukocyte recruitment in TNF-α induced inflammation or thioglychollate induced peritonitis. We should keep in mind, however, that all three selectins co-operate at some level to influence eosinophil homeostasis, whereas P selectin is the only selectin whose absence impairs the recruitment of these cells to the inflamed peritoneum, the combined absence of P- and E-selectins seems to lead to the complete abrogation of the allergic response into the development of an asthma phenotype [34].

However, the conclusive role of L selectin vs. all three is somewhat controversial. Previous works by different groups have presented somewhat disparate results. A study with an anti-L-selectin antibody or L-selectin gene knock out mice could not prevent development of asthma [35]. Other groups have shown that neither L- nor E-selectin contributed to sinusoidal neutrophil sequestration or transmigration. Yet other groups have shown that adoptive transfer of splenic lymphocytes from Cockroach antigen- (CRA) primed E- and P- selectin deficient cells into naive wild-type (WT) mice produced the same level of airway hyperreactivity as transfers from CRA-primed WT into naive WT hosts, indicating similar peripheral immunization and [36] similar serum IgE production the selectin-deficient and WT animals, indicating that the Th2-driven isotype switch was unaffected by the genetic alterations. Thus both P- and E-selectin contribute to CRA-induced peribronchial inflammation and airway hyperreactivity[37].

Exclusive role of L selectin in OVA asthma was shown by the following studies: While OVA sensitized/challenged ICAM-1-deficient mice showed decreased levels of B220+ lymphocytes in the BALf, OVA-sensitized/challenged L-selectin deficient mice demonstrated significantly reduced numbers of CD3+ lymphocytes and increased B220+ lymphocytes in BALf suggesting a crucial role for ICAM-1 in airway inflammation and AHR in asthma (as corroborated by our earlier studies [11,12] but L-selectin plays a more selective role in the development of AHR but independent of airway inflammation in this animal model of asthma [38].

Thus earlier studies have shown that both cell adhesion molecules (i.e. selectins and integrins) play key roles in cell trafficking and in the lung they regulate leukocyte extravasation, migration within the interstitium, cellular activation, and tissue retention. However, adoptive transfer experiments show very clearly that sensitized CD4+ T cells from WT spleens were able to reverse the effect of ELP deletion in developing an asthma phenotype although of a smaller magnitude than sensitized and challenged WT. It follows that CD4+ T cells from WT will express only L-selectin of all the three selectins and therefore L-selectin alone was sufficient to reverse the effects of E-, L- and P- selectin deletion [39].

The significant reduction in levels of all immunoglobulins (Figure 3) indicate a serious problem with Ig sequestering by ELP-/- B cells. If CD62L+CD4+ T cells from sensitized WT spleen could develop asthma in naïve ELP-/-, it also indicates that development of asthma may not be entirely dependent on ELP-/- B cells in the spleens of these ELP-/- recipients or that the 5 million CD62L+ sensitized CD4+T cells are sufficiently potent to go into circulation and generate at least threshold amount of IgE for required airway inflammatory response. As seen from ELISPOT assays (Figure 4) only the axillary lymph nodes showed increase in IL-4+ expressing cells which may have provided the required mobilizing force for the nearby airway inflammation in the adoptively transferred recipients.

The role of selectins in neutrophil trafficking in the lungs was frequently considered negligible since the narrow pulmonary capillaries cannot accommodate the typical selectin-mediated rolling phenomenon. Furthermore, selectins did not seem necessary in lung neutrophil sequestration since the deceleration of circulating neutrophils prior to their firm adherence was effectively achieved by their mechanical retention [40]. Yet a large body of experimental data demonstrating that selectin inhibition (via the use of blocking antibodies or selectin antagonists or transgenic knockout of one or more selectins) frequently protected animals from Acute Lung Injury [17].

In the light of the above data let us try and explain what the status of the various cells playing key role in the onset and establishment of the TH2 inflammatory response to the allergen OVA are likely to be. First, the dendritic cells [41] and alveolar macrophages [42] whose responses are critical for antigen presentation to the naïve T cells, need P- and L-selectin and the β2 integrins and L selctins respectively may be considered. These cells therefore are practically dysfunctional when L-selectins are absent on the KO mice. They are neither capable of processing the signal nor coupling with their other accessories that mediate adhesion in the inflammatory cascade.

If we assume that in the ELP-/- mice, there was no response to the allergen in the first place because the cells themselves were incapable of sequestering the allergen for presentation, the absence of any TH2 response and therefore inflammatory recruitment downstream and the consequent non-manifestation of the composite asthma phenotype may be explained. However, (Table 3) increased number of mature leukocytes and progenitors in BM and circulation of post-OVA ELP-/- mice butinsignificantly so in the airways But elevated in the lung parenchyma particularly of monocytes, macrophages and neutrophils (myeloid population) but no eosinophils and mast cells is somewhat confusing because it seems that the systemic response did happen to the OVA challenge but migration to the tissues was arrested.

The adoptive transfer of only ELP+ CD4+ T lymphocytes reinstating the asthma phenotype indicates conclusively that it was the afferent arm of the Th2 immune response that was affected. However, since human asthma involves a complex interplay of cells, early and late asthma responses sometimes intermingle especially during exacerbations in the chronic phase of allergic asthma hence the assumption that the afferent limb of the immune response was likely affected. Also, since the role of CD4+ T cells in eosinophil recruitment is firmly established [43-45] triple selectin deletion may be held responsible for preferably affecting the afferent arm of the response by giving the OVA-treated mouse such a clean respiratory tree and no hyperreactivity to methacholine.

As for attenuation of both Th1 and Th2 cytokines in the ELP-/- OVA-treated mice, the non-occurrence of the first lap of the journey (the afferent limb of the immune response) probably prevents activation and recruitement of the cytokine secreting cells [46]. The elegant study by David A. Randolph et al [47] show that while both Th1 and Th2 cells are recruited to the airways, Th1 cells predominate early and Th2 cells predominate late. On this assumption we conclude that it is not exclusively the Th2 cells that are recruited and further reduction in Th1 and Th2 cytokine secretion correlates with the complete non-occurrence of the first line response in the development of the composite asthma phenotype in the knockout animals.

In the light of our findings [11,12] with the acute and chronic OVA models, the β2 integrins, which, compared to α4 integrins preferably co-localize with the P- and L- selectins in these cells, there seems to be a disruption of a similar pathway that also prevents development of asthma in the CD18 knockout mice. However, release of Th2 specific cytokines being significantly downregulated in KO BALf and plasma (Table 4) and virtually no IL-4-secreting cells being detected in all tissues systemic as well as local, (Table 4, 5) a universal lack of response in processing the stimulus viz. OVA, is indicated. The cells that were hyperproliferated in bone marrow and blood, were also incapable of sequestering the requisite cytokines to mount a response. Interestingly peripheral lymph nodesrelease higher levels of TH1 cytokines indicating that deletion of all three selectins may be critical for skewing the inflammatory responses towards a TH2 phenotype at least in an acute allergic set up. Whether the situation will reverse in the chronic phase as in our work with the β2 integrin knockout mice [12] is yet to be studied.

This is the first report that clearly delineates the role of L-selectin from the other selectins and show that: (a) ELP-/- mice are incapable of mounting a full fledged asthma response despite impressive peripheral leukocytosis possibly due to a failure to sequester initial response to allergen exposure; (b) inflammatory response in airways is almost non-existent despite some myeloid migration into their lungs; (c) ELP-/- T cells are functionally active and responsive whereas ELP-/- B cells are incapable of sequestering total as well as allergen specific antibody which may indicate (1) that the B cells to be "primed" during initial sensitization by OVA-alum complex may have been ineffective (which is more likely) or (2) the primed B cells when matured into plasma cells fail to properly sequester OVA-specific IgE (which although an attractive explanation, cannot explain how in the adoptive transfer experiments (Figure 7 and Table 5), naïve ELP-/- recipients could successfully mount an asthma response when transplanted with CD4+ WT T cells which could happen only if the sensitization phase occurred successfully. Nevertheless the fact that they were able to manifest "functional asthma" (as shown by lung function test) despite unprimed B cells may indicate that the robust response was due mainly to the CD4+ T cell response that were equipped with the L-selectin and the endothelium of the challenged mouse having their E-selectins intact and lastly, P-selectins on the recipient platlets which may have played a key role in neutrophil sequestration (Table 1, 2 and 3); (d) sensitized CD62L+ CD4+ T cells adoptively transferred into naïve ELP-/- recipients could reverse the effects of ELP deletion and non-development of asthma; and lastly (e) the axillary lymph nodes may be key to mobilize IL-4+ cells/IL-4 for Th2 response and inflammatory recruitment in the airways the significance of which at this point is unclear.

Due to the essential role played by these cell adhesion molecules in lung inflammation, all selectin family members (including L-selectin, P-selectin, and E-selectin) appear to be important therapeutic targets [48]. Indeed, Revotar Biopharmaceuticals AG is developing the drug under license from Encysive, which is a CD62L antagonist (TBC-1269) for the potential treatment of asthma, COPD, VILI, ALI and ARDS.

## Conclusion

E-, L-, and P- selectins are important drug targets for asthma. Locally applicable combination therapies with small molecular antagonists or antibodies may be useful in acute allergic asthma treatment regimens to supplement other conventional therapies.

## Conflict of interest

The authors declare that they have no competing interests.

## Acknoledgements

This work was supported by National Institutes of Health grants (HL58734, DK46557) for Thalia Papayannopoulou. The authors thank Dr. Arthur L. Beaudet for the kind gift of the triple selectin knockout mouse. The authors also thank Gregory Priestley for efficient supervision of the mouse facility and Devra Batdorf for expert mouse handling and fellows of the ERB lab Utpalendu Ghosh, Arijit Ghosh, and Arindam Saha for helping with the edits of this manuscript.

## Abbreviations used

KO: knockout; BALf; bronchoalveolar lavage fluid; LP: lung parenchyma; PB: peripheral blood; AHR: airway hyperresponsiveness; Mch: methacholine; Eos: eosinophils; PMN: polymorphonuclear neutrophils, MNC: mononuclear cells; ICAMs: Intracellular Adhesion Molecules; H&E: Hematoxylin and Eosin; CLN: cervical lymph node; MLN: mesenteric lymph node; LNX: axillary lymph node; LNI: inguinal lymph node; CRA: cockroach allergen; VILI: ventilation induced lung inflammation; ALI: acute lung injury; ARDS: adult respiratory distress syndrome; CFU-c: colony forming units count.
